# What We Talk about When We Talk about Artificial Intelligence in Radiation Oncology

**DOI:** 10.3390/jpm12111834

**Published:** 2022-11-03

**Authors:** Francesco Cuccia, Giuseppe Carruba, Guseppe Ferrera

**Affiliations:** 1 Radiation Oncology, ARNAS Civico Hospital, 90100 Palermo, Italy; 2 Division of Internationalization and Health Research (SIRS), ARNAS Civico Hospital, 90100 Palermo, Italy

The constant evolution of technology has dramatically changed the history of radiation oncology, allowing clinicians to deliver increasingly accurate and precise treatments, moving from 2D radiotherapy to 3D conformal radiotherapy, leading to intensity-modulated image-guided (IMRT-IGRT) and stereotactic body radiotherapy treatments [1,2].

In recent years, we have witnessed an ever-growing attention in the scientific community towards the application of artificial intelligence (AI), with initial experiences that highlight the potential benefits of this technological ally [3,4,5].

Within the definition of AI, there is a wider concept that encompasses all the potential declinations of the aptitude of a machine to mimic human intelligence [6].

In recent decades, the scientific community has been overwhelmed by a dramatically increasing amount of data and information, from both a technological (imaging data) and a biological perspective (DNA and RNA analysis and mutational signatures) [7].

In this scenario, devices endowed with the capacity of speeding up the acquisition and analysis of data are attractive and promising tools for clinicians to provide more tailored and personalized approaches.

Among all medical disciplines, radiation oncology is probably one of the most strictly connected to technological advances. Thus, the potential applications of AI may be implemented across all phases of radiotherapy treatment: from the analysis of diagnostic imaging to identifying radiological features predictive of clinical outcomes, to target auto-contouring or treatment planning automation, eventually leading to on-board imaging analysis to identify potential features predictive of higher toxicity or lower local control rates [8,9,10,11].

With this aim, machines trained to translate radiological features into clinical data may identify predictive and prognostic information that is useful to deeply customize treatment, as exemplified in a study by Cunliffe et al. that investigated radiomics features as potential predictors of radiation pneumonitis after lung radiotherapy, or in the study by Matrone et al., published in this issue, reporting the potential identification of local failure in patients who received partial prostate re-irradiation [12,13].

This Special Issue, entitled “Artificial Intelligence in Radiation Oncology”, aims to collect preliminary experiences of AI-based radiotherapy and present challenges, hopes, and food for thought for future studies.
